# Novel superpixel method to visualize fundus blood flow resistivity in healthy adults

**DOI:** 10.1038/s41598-023-33450-2

**Published:** 2023-04-15

**Authors:** Kenji Okamoto, Noriyoshi Takahashi, Tatsuhiko Kobayashi, Tomoaki Shiba, Yuichi Hori, Hitoshi Fujii

**Affiliations:** 1 Softcare Co., Ltd., Fukutsu, Japan; 2 Department of Ophthalmology, School of Medicine, Toho University, Tokyo, Japan; 3 Department of Ophthalmology, International University of Health and Welfare Narita Hospital, Chiba, Japan; 4 Department of Computer Science and Electronics, Kyushu Institute of Technology, Iizuka, Fukuoka Japan

**Keywords:** Medical imaging, Diagnosis

## Abstract

We aimed to perform superpixel segmentation of ocular blood flow maps obtained using laser speckle flowgraphy (LSFG) and investigate the effects of systemic parameters such as body weight, height, and sex on ocular blood flow resistivity. We studied 757 healthy participants (583 men, 174 women). We calculated the average beat strength over mean blur rate (BOM) as a LSFG resistivity index, as a function of age and sex using ordinary regions of interest (ROI) centered on the optic nerve head (ONH), the retinal vessels region and tissue around the ONH, and the choroid (CHD). We compared the ROI and superpixel-based methods, which are segmented based on image processing, for calculating the BOM. The sex differences in the BOM for the ONH, retinal-vessels region and tissue region of the ONH and CHD were significant for individuals aged ≤ 50 years (*P* < 0.01) but not those > 50 years old (*P* > 0.05). The average BOMs calculated using the ROI and superpixel methods were strongly correlated in the ONH (coefficient = 0.87, *R*^2^ = 0.8, *P* < 0.0001,* n* = 5465). In summary, a superpixel-segmented BOM map is suitable for two-dimensional visualization of ocular blood flow resistivity.

## Introduction

Laser speckle flowgraphy (LSFG) is a non-invasive and quantitative method^1^ for determining ocular blood flow^2,3^. We previously studied the relationship between blood flow resistivity and aging in healthy adults using LSFG^4^, with the aim of estimating age based on ocular blood flow measurement during a health check or complete physical examination. LSFG monitors the movement of erythrocytes in the retina, choroid (CHD)^5^, and optic nerve head (ONH), and the mean blur rate (MBR) is used as an indicator of ocular blood flow^6,7^. Several studies have revealed that age^8–11^ and sex^8–12^ are significantly correlated with the average MBR in the ONH. The change in MBR throughout the cardiac cycle is represented by waveform parameters such as blowout score (BOS)^13–18^ and blowout time (BOT)^14,19–25^, which are used to quantify age-dependent increases in resistivity^8^ and to assess the relationship between blood flow and resistivity^26^.

BOS and BOT partly reflect vascular resistance^27,28^, but their calculation requires averaging from separate images acquired for each heartbeat, and these parameters are thus strongly influenced by heart rate. Beat strength (BS) over the temporal average of the MBR (BOM) parameter, which does not depend on heart rate, was therefore devised to overcome this drawback. The BOM is an LSFG waveform parameter obtained from frequency analysis and is similar to the pulsatility index (PI) used in the Doppler method to detect the resistivity of blood flow in systemic vascular disease^29,30^. Like BOT and BOS^18^, the BOM parameter is a measure of vascular resistance, with the additional advantage of not requiring the detection of separating heartbeats. The BOM can also be applied to the relative flow volume (RFV) parameter^31^, to assess resistivity in retinal and choroidal vessel segments.

Vascular resistance is a key factor in the pathogenesis of retinal vein occlusion (RVO), which is a common retinal vascular disease^32,33^. It is important to identify the location of the vascular occlusion in patients with vascular occlusive diseases, such as central RVO (CRVO) and branch RVO (BRVO), but it is difficult to determine the location of the increased vascular resistance using blood flow maps alone. Until now, a single BOM value has been calculated from time-series blood flow values by drawing a region of interest (ROI), known as a ‘rubber band’, around the vessel with the suspected vascular occlusion^34^. This rubber band used in LSFG can be a rectangle, ellipse, or other shape. In fundamental analysis, the average values of the parameters within the ROI enclosed by the rubber band are calculated and used as representative values for that region. However, it would be clinically useful to automatically map the vascular resistance before the rubber band is placed, to enable the location of the vascular occlusion to be easily identified.

**Figure 1:**
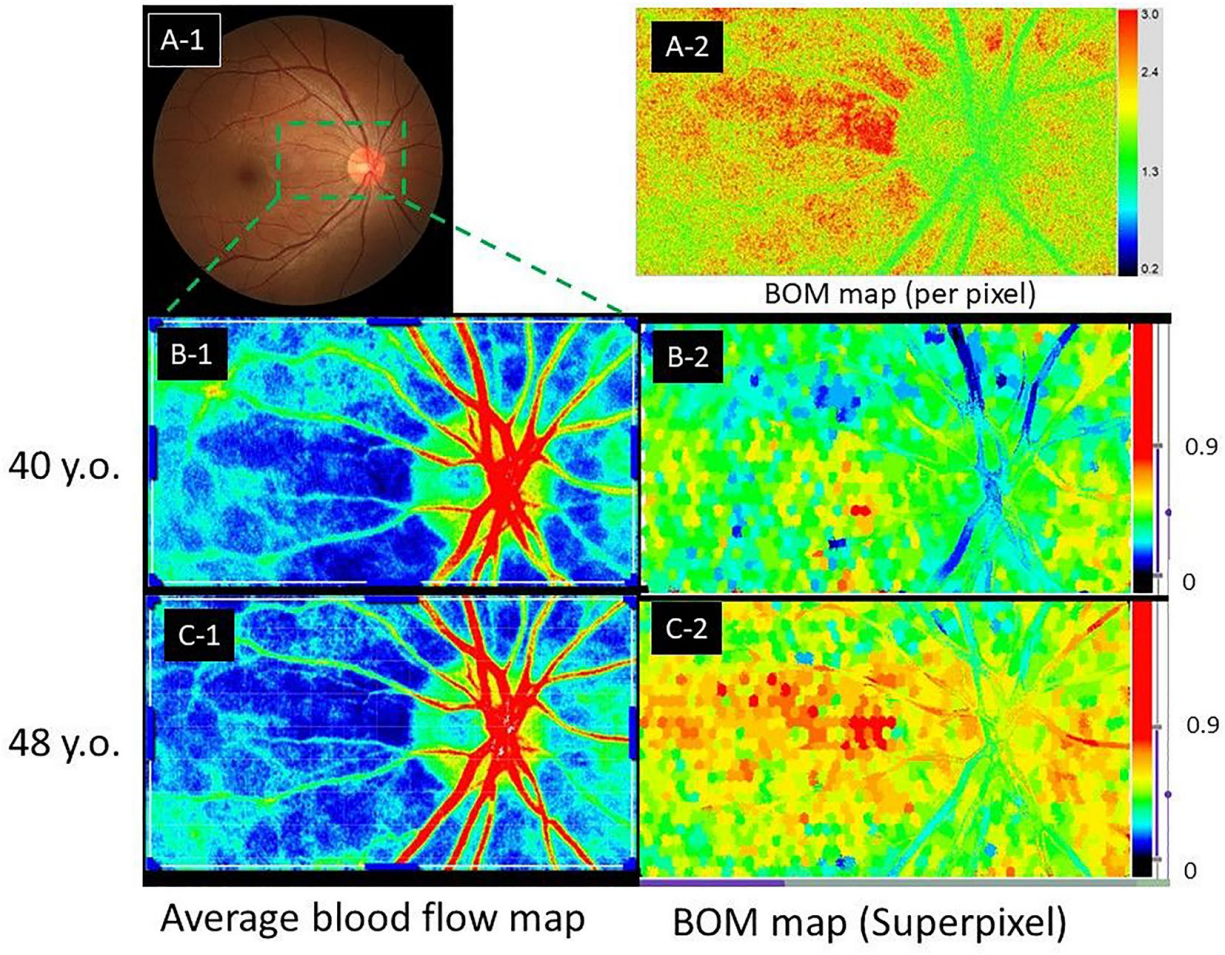
Example of superpixel-segmented BOM maps. Representative maps of average blood flow and resistivity (BOM) obtained using laser speckle flowgraphy and simple linear iterative clustering. Images show the same healthy subject at ages 40 and 48 years. The average blood flow maps show no discernable change (cf. panels **B-1** and **C-1**), but the BOM maps show an age-dependent increase in retinal blood vessel resistivity and choroidal resistivity (cf. panels **B-2** and **C-2**). (**A-1**) is the fundus image of the same eye. (**A-2**) is a pixel-wise BOM map. BOM, average beat strength over mean blur rate; y.o., years old.

**Figure 2:**
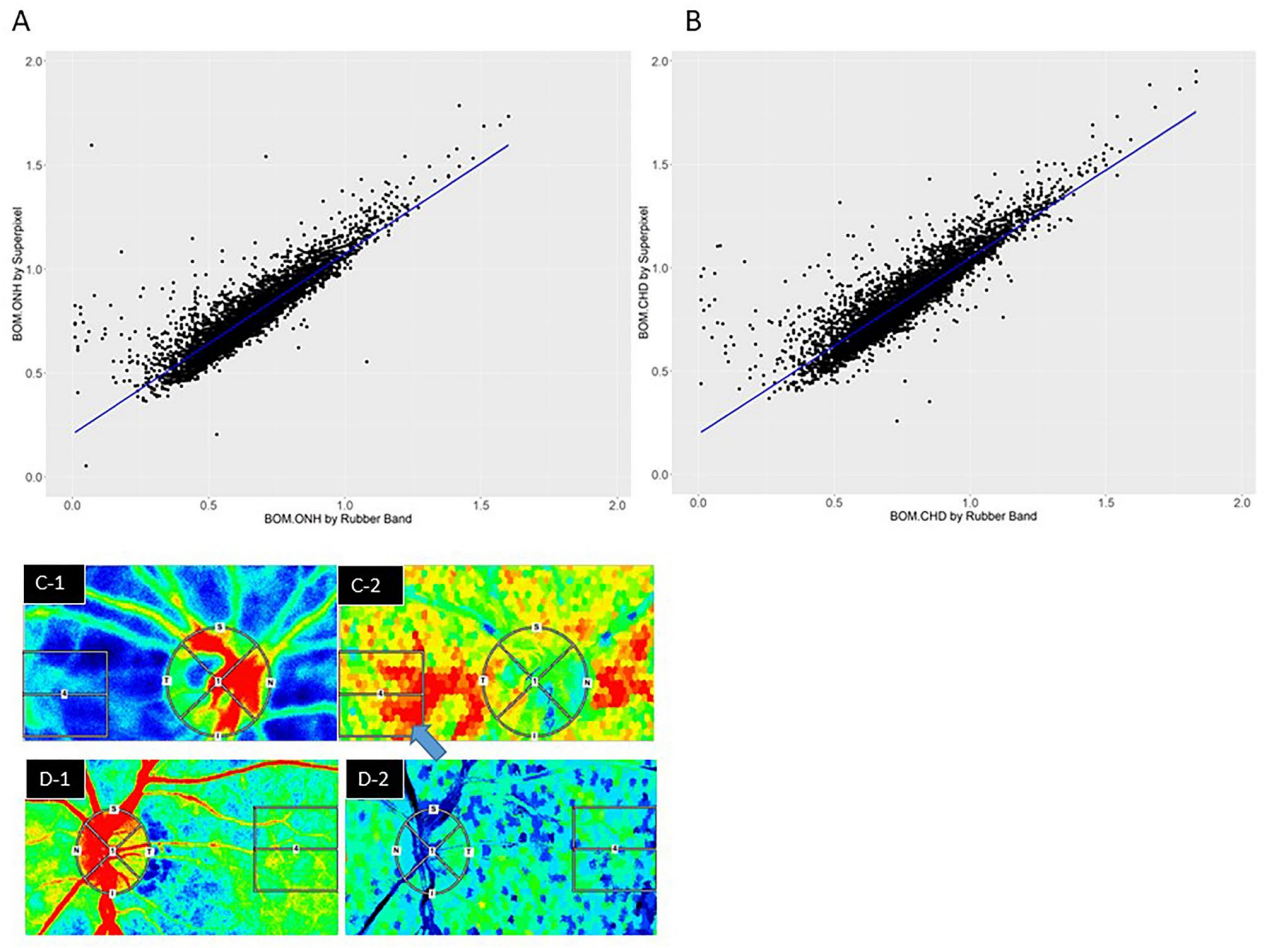
Scatter plots of BOM, (**A**) Optic nerve head (BOM.ONH) and (**B**) choroid (BOM.CHD), calculated using the rubber band method and the superpixel method. These scatter plots do not include BOM.T or TCR. The blue line on each graph was fitted using univariate regression. There were good correlations between the parameters calculated using the rubber band method and superpixel method for both regions (ONH: *R*^2^ = 0.8, CHD: *R*^2^ = 0.81). (**C-1**) is the fundus image of the same eye. Examples of average blood flow maps and BOM maps that showed high correlation (**C-1**,**C-2**) between the rubber band method and the supperpixel method are shown in panels (**C-1**) and (**C-2**); maps with low correlation are shown in (**D-1**) and (**D-2**). The blue arrow in (**C-2**) indicates an artifact caused by a shadow of the eyelash present during measuring.

Recent improvements in image analysis software have made it possible to visualize superpixel-segmented BOM maps^34^ as blood flow maps (Fig. 1). The fully automated superpixel segmentation method reduces computation time by treating multiple pixels as a single cluster, which enables better visualization of blood flow^35,36^. Pixel-wise calculation and mapping of the BOM is possible but visually complex (Fig. 1A-2), time-consuming, and unsuitable for clinical use. For example, the pixel-wise computation time for one measured set of LSFG images used in this study was 15,795 s (4.4 h), compared with a corresponding computation time of 10 s using the superpixel method. In addition, superpixel segmentation attenuates the speckle noise in the LSFG blood flow map, making the structural boundaries clearer. However, one disadvantage is that the BOM values obtained using the superpixel method are higher than those obtained using the standard rubber band method, and the BOM thresholds for vascular occlusion obtained with the standard rubber band method in previous studies therefore cannot be used.

Regarding the threshold of BOM for vascular occlusion, Matsumoto et al. reported that the total capillary resistance (TCR), defined as the BOM in the major vessels (arteries and veins) in the ONH, was higher for CRVO patients than for normal subjects, irrespective of sex and age^37^, while Yanagida et al. reported a sex-based difference between resistance values in normal subjects^11^. However, these reports did not elaborate on the association with systemic parameters such as body weight, height, or ocular perfusion pressure (OPP). The first aim of this study was to clarify the relationship between BOS and BOM as a measure of resistivity. The second aim was to assess the systemic parameter- and sex-based differences in BOM values in the ONH, retinal vessels, and CHD in healthy adults. The third aim was to visualize superpixel-segmented BOM maps by averaging their superpixel intensities and compare them with a threshold value, to detect blockages in patients. A clear relationship between the average BOM values of multiple superpixels and the BOM values obtained using the standard rubber band method would make it possible to determine by inspection if the BOM map was normal.

## Results

### Relationship between BOS and BOM

A total of 325 of the 1082 participants were excluded, leaving 757 participants (583 men, 174 women) who met the study criteria. A total of 514 participants were ≤ 50 years old (393 men, 121 women) and 243 were > 50 years old (190 men, 53 women). We used univariate regression analysis to determine the associations between BOS and BOM in the ONH (r =  − 0.95, *P* < 0.001), retinal vessels (r =  − 0.92, *P* < 0.001), the tissue surrounding the vessels (r =  − 0.96, *P* < 0.001) and in the CHD (r =  − 0.84, *P* < 0.001).

### Characteristic of BOM in healthy adults based on systemic parameters

Table 1 shows the systemic parameters and BOM values for the entire ONH (BOM.A), CHD (BOM.CHD), retinal vessels (TCR^37^), and the tissue surrounding the vessels in the ONH (BOM.T) in relation to sex and age.

**Table 1:** Parameters of participants included in this study.

Parameters	≦ 50 y		> 50 y	
	Men/women	P	Men/women	P
n	393/121		190/53	
Age, yrs	43.6 ± 4.6/42.9 ± 4.9	0.181	57.2 ± 5.7/57.4 ± 5.4	0.424
Height, cm	172.0 ± 5.7/160.1 ± 4.6	< 0.001***	169.4 ± 5.9/156.6 ± 5.5	< 0.001***
Weight, kg	71.2 ± 11.5/56.4 ± 9.4	< 0.001***	68.2 ± 9.4/55.2 ± 10.0	< 0.001***
Heart rate, bpm	70.8 ± 10.6/71.9 ± 9.2	0.255	69.5 ± 9.5/70.0 ± 10.2	0.920
SBP, mmHg	122.0 ± 17.0/113.1 ± 15.1	< 0.001***	127.7 ± 19.6/122.9 ± 18.3	0.104
DBP, mmHg	76.4 ± 12.1/68.0 ± 10.9	< 0.001***	80.2 ± 13.0/72.9 ± 11.4	< 0.001***
MABP, mmHg	91.6 ± 13.1/83.0 ± 11.6	< 0.001***	96.1 ± 14.6/89.5 ± 12.6	< 0.01**
IOP, mmHg	12.0 ± 2.4/11.7 ± 2.4	0.180	11.8 ± 2.8/11.8 ± 2.3	0.75
OPP, mmHg	49.0 ± 8.7/43.5 ± 7.5	< 0.001***	52.3 ± 9.9/47.9 ± 8.5	< 0.01**
BOM parameters				
TCR	0.49 ± 0.21/0.60 ± 0.24	< 0.001***	0.56 ± 0.28/0.59 ± 0.27	0.057
BOM.A	0.59 ± 0.20/0.69 ± 0.23	< 0.001***	0.67 ± 0.27/0.71 ± 0.26	0.046*
BOM.T	0.68 ± 0.21/0.78 ± 0.24	< 0.001***	0.78 ± 0.29/0.82 ± 0.30	0.059
BOM.CHD	0.70 ± 0.15/0.78 ± 0.14	< 0.001***	0.82 ± 0.20/0.85 ± 0.18	0.214

Data (mean ± standard deviation) were compared by sex (Wilcoxon’s rank sum test). IOP, intra-ocular pressure; MABP, mean arterial blood pressure; OPP, ocular perfusion pressure; sex-dependent resistivity of blood flow (BOM) in the optic nerve head (BOM.A), retinal vessels (TCR), choroid (BOM.CHD), and non-vessel tissue in the optic nerve head (BOM.T). *P < 0.05; **P < 0.01; ***P < 0.001.

We performed multiple regression analysis to compare BOM parameters and sex, weight, height, and OPP between the two age groups (Table 2). Sex and OPP showed strong correlations (*P* < 0.001) with all BOM parameters in participants ≤ 50 years, and BOM.A and BOM.T were significantly correlated (*P* < 0.05) with weight and height. In participants > 50 years, TCR was correlated with OPP (*P* < 0.05) and was strongly correlated (*P* < 0.01) with weight, and BOM.A and BOM.T were correlated (*P* < 0.05) with weight.

**Table 2:** Results of multiple linear regression analysis, β: Standardized partial regression coefficient. BOM parameters, including resistivity of blood flow (BOM) in the optic nerve head (BOM.A), retinal vessels (TCR), choroid (BOM.CHD), and non-vessel tissue in the optic nerve head (BOM.T) were response variables. SEX (Woman:0, Men:1), OPP, ocular perfusion pressure.

BOM parameters	Aged group	Term	β (95% CI)	Standard error	Statistic	P
TCR	≦ 50 y	SEX	−0.7460 (−1.0100 to −0.4860)	0.1330	−5.6200	< 0.001***
		Weight	−0.0632 (−0.1680 to 0.0415)	0.0533	−1.1900	0.24
		Height	0.1010 (−0.0151 to 0.2170)	0.0590	1.7100	0.09
		OPP	−0.2410 (−0.3290 to −0.1520)	0.0451	−5.3400	< 0.001***
	> 50 y	SEX	−0.0489 (−0.4600 to 0.3620)	0.2080	−0.2340	0.82
		Weight	−0.2340 (−0.4050 to −0.0639)	0.0865	−2.7100	< 0.01**
		Height	0.1200 (−0.0743 to 0.3150)	0.0988	1.2200	0.22
		OPP	−0.1490 (−0.2820 to −0.0150)	0.0678	−2.1900	0.03*
BOM.A	≦ 50 y	SEX	−0.7500 (−1.0100 to −0.4910)	0.1320	−5.7000	< 0.001***
		Weight	−0.1290 (−0.2330 to −0.0257)	0.0528	−2.4500	0.01*
		Height	0.1360 (0.0213 to 0.2510)	0.0585	2.3300	0.02*
		OPP	−0.2300 (−0.3170 to −0.1420)	0.0447	−5.1400	< 0.001***
	> 50 y	SEX	−0.0832 (−0.4950 to 0.3290)	0.2090	−0.3980	0.69
		Weight	−0.2160 (−0.3870 to 0.0447)	0.0868	−2.4900	0.01*
		Height	0.1000 (−0.0951 to 0.2960)	0.0991	1.0100	0.31
		OPP	−0.1340 (−0.2680 to 0.0002)	0.0681	−1.9700	0.05
BOM.T	≦ 50 y	SEX	−0.7430 (−1.0000 to −0.4810)	0.1330	−5.5700	< 0.001***
		Weight	−0.1320 (−0.2370 to −0.0273)	0.0535	−2.4700	0.01*
		Height	0.1450 (0.0285 to 0.2610)	0.0593	2.4500	0.01*
		OPP	−0.2020 (−0.2910 to −0.1130)	0.0453	−4.4600	< 0.001***
	> 50 y	SEX	−0.0661 (−0.4810 to 0.3490)	0.2110	−0.3140	0.75
		Weight	−0.1800 (−0.3520 to −0.0076)	0.0875	−2.0600	0.04*
		Height	0.0586 (−0.1380 to 0.2550)	0.0999	0.5860	0.56
		OPP	−0.1030 (−0.2380 to 0.0322)	0.0686	−1.5000	0.14
BOM.CHD	≦ 50 y	SEX	−0.4530 (−0.7280 to −0.1780)	0.1400	−3.2400	< 0.01**
		Weight	−0.0942 (−0.2050 to 0.0165)	0.0564	−1.6700	0.10
		Height	0.0445 (−0.0782 to 0.1670)	0.0625	0.7130	0.48
		OPP	−0.1300 (−0.2240 to −0.0361)	0.0478	−2.7200	< 0.01**
	> 50 y	SEX	−0.1790 (−0.6010 to 0.2440)	0.2140	−0.8330	0.41
		Weight	−0.0077 (−0.1830 to 0.1680)	0.0892	−0.0866	0.93
		Height	0.0695 (−0.1310 to 0.2700)	0.1020	0.6820	0.50
		OPP	−0.1070 (−0.2440 to 0.0298)	0.0695	−1.5400	0.13

### Comparison of BOM values calculated using rubber band and superpixel methods

In this study, we attempted to acquire data three times per eye for both eyes of all 1082 healthy participants. Some eyes were difficult to measure and, even if they could be measured, the rubber band could not be set due to poor fixation. As a result, 2004 eyes were measured, and rubber bands were set on the data that measured well, so a total of 5465 data were included in this analysis. Scatter plots of BOM (*n* = 5465) in the ONH (Fig. 2A) and CHD (Fig. 2B) were analyzed by linear regression. Significant correlations were identified for the ONH (coefficient = 0.870, *R*^2^ = 0.80, *P* < 0.001) and CHD (coefficient = 0.852, *R*^2^ = 0.81, *P* < 0.001). For the ONH, the linear relationship was:

1 $${\text{BOM}}.{\text{ONH}}_{{{\text{SP}}}} = \, 0.{2}0{6 } + \, 0.{87}0 \times {\text{ BOM}}.{\text{ONH}}_{{{\text{RB}}}} ,$$

where the _SP_ and _RB_ refer to the values determined using the superpixel and rubber band methods, respectively. The corresponding relationship for the CHD was:

2 $${\text{BOM}}.{\text{CHD}}_{{{\text{SP}}}} = \, 0.{196 } + \, 0.{852 } \times {\text{ BOM}}.{\text{CHD}}_{{{\text{RB}}}} .$$

Shown in Fig. 2 are examples of average blood flow and BOM maps with high correlation (Fig. 2C-1, C-2) and low correlation (Fig. 2D-1, D-2) between BOM.ONH_RB_ and BOM.ONH_SP._ The superpixel structures of the BOM in the CHD and ONH tissue areas changed significantly, possibly because of changes in the distribution of blood.

The coefficients of variation, as measures of repeatability, were 5.7 ± 4.0% (superpixel) and 8.7 ± 6.9% (rubber band) in the ONH, and 6.1 ± 4.3% (superpixel) and 8.7 ± 7.1% (rubber band) in the CHD.

## Discussion

Our previous study showed that women had a lower BOS across the entire ONH than men^4^. A low BOS is associated with high resistivity^38^, and given that BOM is considered to be the inverse property of BOS^4^, a high BOM value corresponds to high resistivity. We showed a significant correlation between BOS and BOM, thus indicating that BOM represents the resistivity value in each region.

The resistivity value in each region depended on sex, weight, height, and OPP in participants aged ≤ 50 years. Height was positively correlated with BOM.A and BOM.T in participants ≤ 50 years old. However, there was no significant correlation to resistivity values in participants > 50 years old. Weight affected resistivity in each region, and OPP and sex had significant impacts on resistance values. Regarding the higher resistivity in women, one of the reasons may be functional, as the artery compliance of the retina is significantly lower in women than in men and blood flow is faster. The greater volume of blood flowing through lower vessels results in higher resistivity. In fact, it has been reported that the elasticity of small arteries is significantly lower and systemic vascular resistance (SVR) is higher in young healthy women than in men^39^. Another report referred to mechanisms by which estrogen reduces vasoconstriction^40^. These earlier results match our findings. Another study reported a decrease in vascular compliance in women older than 50 years^41^. We believe that premenopausal women maintain vascular compliance by reducing vasoconstriction due to the effects of female hormones, and that such women have the robustness to maintain blood flow in the circulatory system even with high resistance. Decreased resistance can be interpreted as an increased ease of flow. One situation that may facilitate flow is an increase in the total cross-sectional area of capillaries. This is equivalent to an increase in the number of capillaries. Since the number of capillaries is thought to increase with increasing body weight, resistance and body weight may be negatively correlated. OPP is the effective intraocular return pressure, so a decrease in resistance will result in an increase in OPP due to easier flow, leading to a negative correlation between resistance and OPP. There were significant differences in height and weight in relation to sex in the different age groups, because the absolute value of the standardized partial regression coefficient for sex was higher than for the other parameters. The BOM.A value for women was higher than that for men up to the age of 50 years, indicating that women had higher ocular blood flow resistivity in the ONH than men in the same age group, consistent with our previous findings^4^. However, there were no significant sex-based differences in BOM.A, BOM.T, or TCR in participants over the age of 50 years. The reduced effect of sex on BOM values as a function of age may be largely related to the menopause. Iwase et al. suggested that the sex hormone estrogen was associated with sex-based differences in blood flow and resistance^10^. Cardiovascular disease is also common in men, but its prevalence in women becomes similar to that in men after the menopause^42^. However, the occurrence of BRVO and retinal artery emboli do not differ between the sexes^43,44^. A different paper reported that women have a higher incidence of systolic hypertension^45^. Additionally, ocular blood flow and kidney damage have a strong relationship^46^. Glycosylated hemoglobin A1c contributed independently to the choroid MBR with a negative correlation between each other^47^. Therefore, peripheral vascular resistance is greater in women, and peripheral organ damage, such as that caused by ocular macrovascular and microvascular diseases, may therefore require more systemic management, including maintenance of suitable blood pressure, in women. These findings may help to clarify the effect of sex on the development of these macrovascular and microvascular diseases.

In clinical studies of CRVO^37,48^, measurement of TCR in addition to MBR enabled threshold TCR values to be established for the presence (0.93) and absence (0.68 ± 0.20) of CRVO. We calculated TCR values of 0.56 ± 0.28 for men and 0.59 ± 0.27 for women in participants > 50 years old, suggesting the absence of CRVO in our cohort. Because the systemic parameter- and sex-based differences in this age group were much smaller than the increase in TCR required to reach the threshold for CRVO diagnosis, our findings also supported the proposal that the TCR threshold does not depend on sex^37,48^.

As for comparison of rubber band and superpixel methods of BOM calculation,our data identified a good correlation between the BOMs calculated using the superpixel and rubber band methods. As for the reproducibility of BOMs, in general, the reliable reproductivity of the COV was less than 10%. This study's results by both methods were acceptable because they were less than 10%. The superpixel images maintained the edges of the retinal vessels because the methods were based on assembling a similar MBR. For both methods, the blood flow in the CHD measured using LSFG was 92% of the average MBR, as previously reported^3,49^, with the BOM value obtained using the rectangular rubber band dominated by the choroidal blood flow. Retinal blood flow in the CHD region enclosed within this rubber band was therefore not expected to affect the measurement of average blood flow value. The BOM values in the CHD region including retinal vessels also had little effect on the measured retinal blood flow, possibly indicating resistance to choroidal blood flow through medium and large vessels.

The intercepts obtained from linear regression analyses showed that the superpixel-derived BOM values in the ONH and CHD regions were approximately 0.2 units higher than those derived using the rubber band method. This discrepancy appears to result from different averaging of the BS, which was calculated from the time-average of many more pixels using the rubber band method compared with the superpixel method. Blood flow data includes a lot of noise caused by unrelated biological signals. The BS measured using the rubber band method is expected to be lower than that determined using the superpixel method because the rubber band method smooths noise in the BS by averaging many data points. For the CHD, each rubber band has 40,000 samples, whereas each superpixel has approximately 200 samples. The variance of blood flow values including speckle noise in LSFG images was unbiased when the number of samples was > 1000 using the rubber band method; speckle noise granularity remained if the number of samples was < 1000. The spatial mean therefore did not converge as the number of samples in the superpixel method increased, resulting in bias. These observations can be described by:

3 $$\begin{aligned} a_{i} & = a_{unbias\_i} + \Delta a_{i} , \quad a_{i} ,a_{unbas\_i} ,\Delta a_{i} \ge 0 \\ b_{i} & = b_{unbas\_i} + \Delta b_{i} , \quad b_{i} ,b_{unbas\_i} ,\Delta b_{i} \ge 0, \\ \end{aligned}$$

where* a*_*i*_, *b*_*i*_ are biased Fourier coefficients of the power spectrum, *a*_unbias_*i*_ and *b*_unbias_*i*_ are their corresponding biased values, and their differences are given by Δ*a*_*i*_ and Δ*b*_*i*_, respectively. In the case of superpixels, substituting Eqs. (3) into Eq. (12) in the Methods section yields:

4 $$\begin{aligned} {\text{Pss}}\left( {f_{i} } \right) & = \sqrt {\left( {a_{unbias\_i} + \Delta a_{i} } \right)^{2} + \left( {b_{unbias\_i} + \Delta b_{i} } \right)^{2} } \\ & = \sqrt {a_{unbias\_i}^{2} + 2a_{unbias\_i} \Delta a_{i} + \Delta a_{i}^{2} + b_{unbias\_i}^{2} + 2b_{unbias\_i} \Delta b_{i} + \Delta b_{i}^{2} } . \\ \end{aligned}$$

The superpixel method averages *n* superpixels, so:

5 $$\begin{aligned} \frac{1}{n}\mathop \sum \limits_{j}^{n} {\text{Pss}}\left( {f_{i} } \right) & = \frac{1}{n}\mathop \sum \limits_{j}^{n} \sqrt {a_{{unbias_{i} }}^{2} + 2a_{{unbias_{i} }} \Delta a_{i} + \Delta a_{i}^{2} + b_{{unbias_{i} }}^{2} + 2b_{{unbias_{i} }} \Delta b_{i} + \Delta b_{i}^{2} } \\ & = \frac{1}{n}\mathop \sum \limits_{j}^{n} \sqrt {a_{{unbias_{i} }}^{2} + \Delta a_{i}^{2} + b_{{unbias_{i} }}^{2} + \Delta b_{i}^{2} } . \\ \end{aligned}$$

Because the values *a*_unbias_*i*_ and *b*_unbias_*i*_ are unbiased and can be considered elements of the rubber band method, the following inequality holds:

6 $$\frac{1}{n}\sum_{j}^{n}\mathrm{Pss}\left({f}_{i}\right)\ge \mathrm{Prb}\left({f}_{i}\right),$$

where Pss and Prb are the power spectra of the superpixel and rubber band methods, respectively. The BS of the superpixel method is therefore greater than that of the rubber band method:

7 $$\mathrm{BSss}\ge BS\mathrm{rb},$$

and this difference causes an offset of the BS. This may be understood by considering that the BS of a biased sample contains noise, so its deviation will be higher. As result:

8 $$\mathrm{BOMss}\ge BOM\mathrm{rb}.$$

The BS calculated using the superpixel method contains more bias; by invoking the concept of noise power, we estimated that this noise contributes an additional offset of approximately 0.2 units to the BOM, as indicated by the intercept factor in Eq. (1) or (2). The method-dependent difference in BOM values may also be caused by differences in the occupation rate between vessels and tissue and in the statistical distributions of the BS and average MBR values, which in turn depend on the sample size. The scatter plots in Fig. 2 show some outliers in the low BOM region, where BOM.ONH_RB_ was < 0.2 and BOM.ONH_SP_ was > 0.5. However, the regression line was not significantly altered after repeat analysis excluding these outliers, and the intercept and slope only decreased by approximately 0.05 units, respectively. Although there was a slight difference between the BOM values derived using the superpixel and rubber band methods, we suggest that superpixel-segmented resistivity maps of the ONH and CHD regions are suitable for quantifying changes in local resistivity associated with diseases such as CRVO, BRVO, and aging.

This study had some major limitations. First, confirmation of the participants’ systemic status, such as their medical history, was only obtained by interview, and the possibility that asymptomatic or systemic disease was present in some participants thus cannot be ruled out. Second, we did not record menopausal status and did not measure levels of sex hormones (estrogen and androgen). Finally, all the participants were Japanese, and it is unclear if our conclusions also apply to non-Japanese populations.

Using LSFG, we identified age- and sex-based differences in the ocular blood flow resistivity parameter BOM in the ONH, retinal vessels, non-vessel tissue in the ONH, and CHD. There were sex-based differences in BOM among individuals ≤ 50 years old, but not in those aged > 50 years; notably, the sex-based difference in the retinal vessels was much smaller than the increase in BOM required to reach the threshold value for a diagnosis of CRVO, supporting previous assertions that this threshold does not depend on sex. We also identified a strong correlation between the BOM values determined using the superpixel and rubber band methods, indicating that superpixel segmentation is a suitable method for visualizing two-dimensional resistivity maps.

## Methods

### Participants

We enrolled healthy 1082 Japanese participants who participated in a medical checkup program at the Department of Health Care Center of the Japan Community Health Care Organization, Tokyo Kamata Medical Center, between December 2016 and December 2018. Participants were excluded if they had any of the following: atherosclerotic disease, such as hypertension, dyslipidemia, diabetes mellitus, cardiovascular or cerebrovascular events, and arrhythmia; ophthalmic disease, such as glaucoma, uveitis, optic neuropathy, vitreous or retinal disease; retinal or choroidal vascular disease; best corrected visual acuity < 40/50; and previous intraocular surgery. Blood pressure measurements and LSFG were performed after patients had rested for 10 min in a quiet, air-conditioned room maintained at 24 °C. All participants abstained from smoking, alcohol, and caffeine for ≥ 24 h prior to the measurements. All evaluations were performed between 9:00 and 11:00 a.m. after the participants had fasted overnight.

### Examinations of the ONH and CHD using LSFG

**Figure 3:**
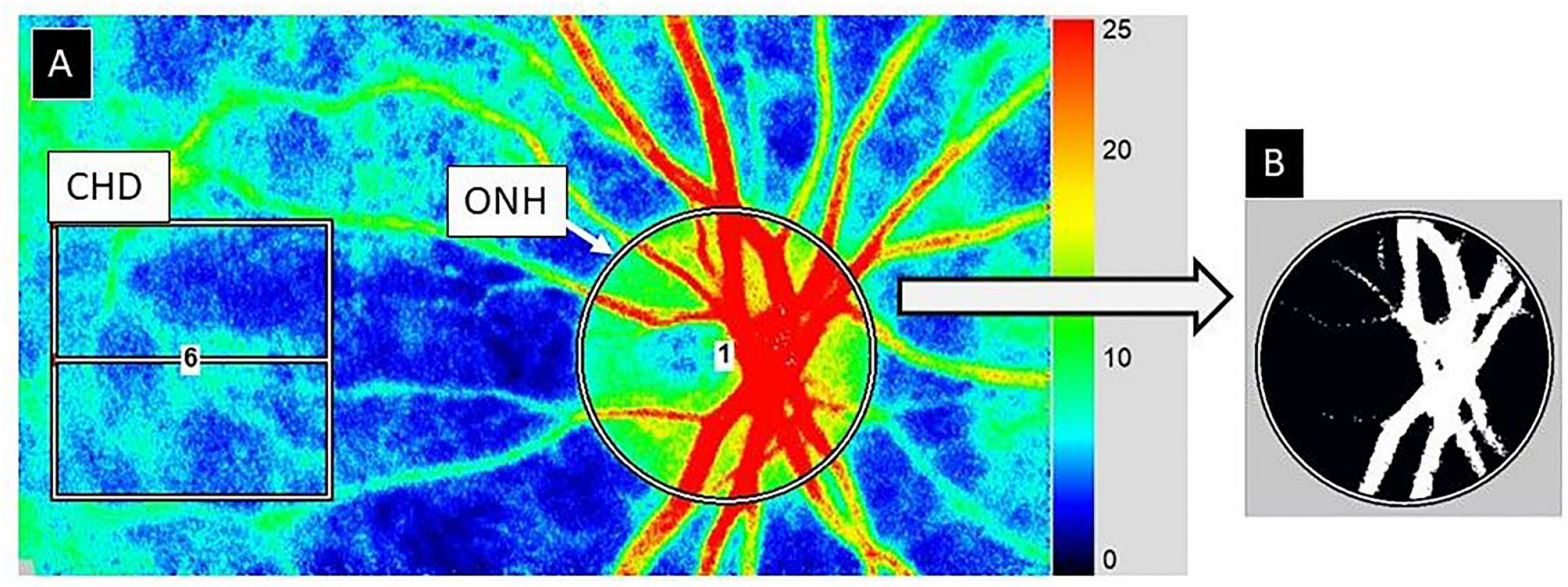
Example ROI, regions of BOM analysis using LSFG. (**A**) Color map of total measurement area. An elliptical rubber band enclosed the entire optic nerve head (ONH) and a rectangular rubber band enclosed a region in the choroid (CHD). The ellipse surrounds the area of the ONH. (**B**) Software was used to demarcate the retinal vessels using an automated definitive threshold throughout the ONH, within the ONH vessels (shown in white) and the ONH tissue (black).

LSFG images were obtained using an LSFG-NAVI system (Softcare Co., Fukutsu, Japan) and the BOM values were calculated using LSFG Analyzer software (V3.8.0.4) running on a Windows-based PC with an AMD Ryzen 7 5800X 8-core processor (3.8 GHz, 32 GB RAM). Briefly, the LSFG system consisted of a fundus camera equipped with a diode laser (wavelength 830 nm) and a CCD camera. For evaluation of the ONH circulation, an elliptical rubber band was placed around the ONH (Fig. 3A). The software delineated the ONH vessels and tissue using an automated definitive threshold with a histogram method (Fig. 3B). The BOM.A parameter was calculated using the BOM in the entire ONH, BOM.T was calculated using the BOM in tissue surrounding the vessels in the ONH, and the TCR was calculated using the BOM in the retina vessels in the ONH.

To evaluate CHD circulation, a rectangular rubber band (200 × 200 pixels) was placed on the temporal side approximately one ONH diameter away from the ONH or as far away as possible, avoiding large retinal vessels (Fig. 3A).

All rubber bands were placed semi-automatically using auto-alignment software (Cobitos V1.0.58.0) with representative rubber bands placed manually by one experienced examiner. All measurements were made with the participants in the seated position, without the use of pupil-dilating eye drops.

### Calculation of BOM to assess the relationship BOS and BOM (first aim)

The amplitude of blood flow in the cardiac cycle divided by the average blood flow defines the resistivity of blood flow quantified by the PI^50^ in Doppler ultrasound. On the basis of the above definition of the PI, we proposed that BOM was an equivalent resistivity parameter in LSFG.

**Figure 4:**
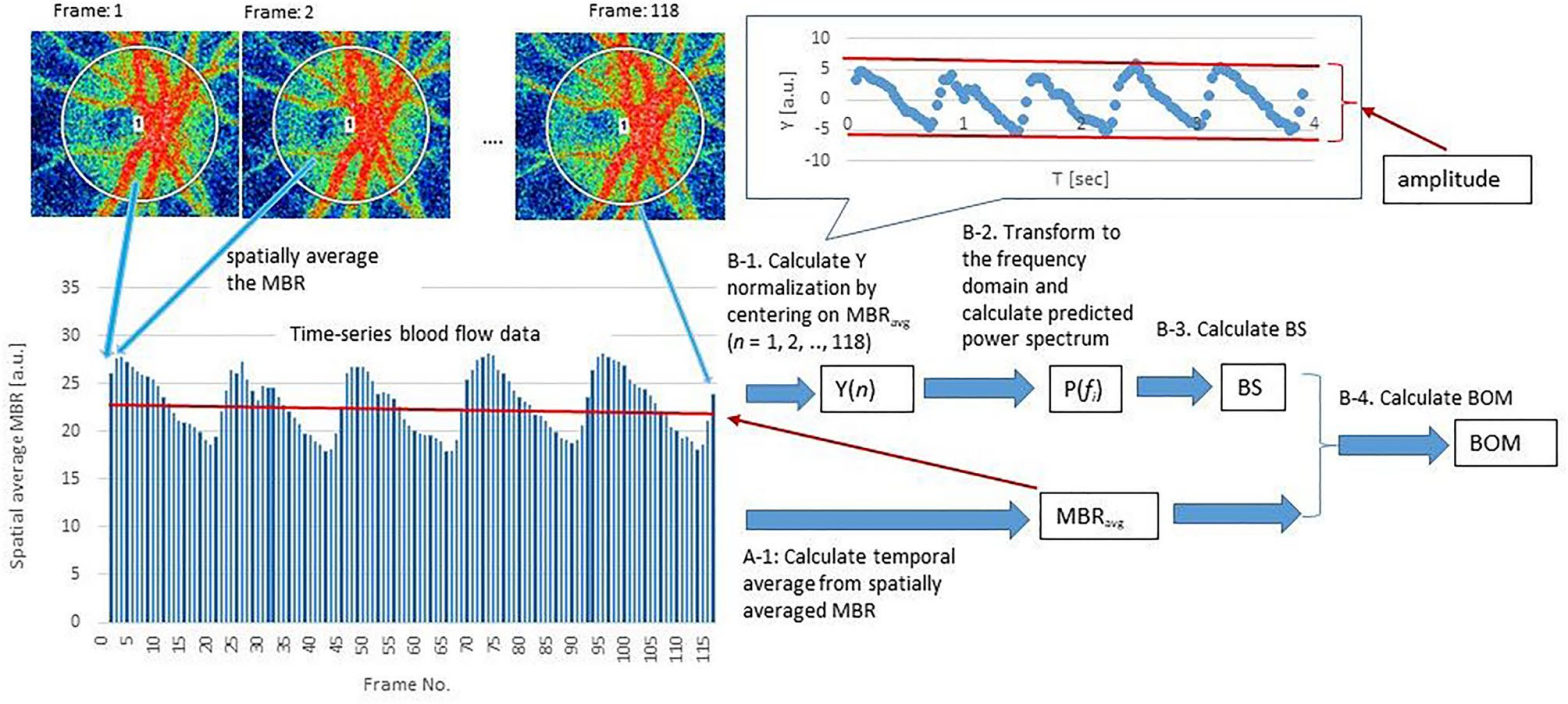
Diagram showing BOM calculation using standard rubber band selection method, representative example using an elliptical rubber band enclosing the entire ONH is shown. Functions Y and P are defined in Eqs. (1) and (4), respectively. MBR, mean blur rate; MBR_avg_, temporal average of the MBR; BS, beat strength.

Using LSFG, the MBR, which is the spatial average of the blur rate measured within the region enclosed by the rubber band, can be captured in 118 frames over a 4-s period and tuned to the cardiac cycle. The amplitude of changes in the MBR can then be used to detect peak-to-peak blood flow. Notably, the BS in LSFG is proportional to the peak-to-peak blood flow and is based on the predicted Fourier spectrum, which, unlike BOS and BOT, does not require heartbeat detection. The predicted Fourier spectrum was calculated using time–frequency analysis based on sparse modeling, and generated a dynamic index describing the strength of heartbeats. BS was defined as the square root of the power of the frequency at the point where the maximum power was observed. Figure 4 illustrates the procedure for obtaining BOM using the standard rubber band method. MBR_avg_ was first calculated as the temporal average of the MBR in the region enclosed by an elliptical rubber band (Fig. 4A-1). The time-series blood flow data Y = (*y*_1_, *y*_2_,…, *y*_118_) were then normalized by centering on MBR_avg_ (Fig. 4B-1). In general, Y can be obtained from a Fourier series expansion of the time information T(*t*_1_, *t*_2_,…, *t*_118_):

9 $$\mathbf{Y}=\mathbf{F}\mathbf{X},$$

where

10 $$\mathbf{F}=\left(\begin{array}{c}\begin{array}{c}\begin{array}{cc}\begin{array}{ccc}\mathrm{sin}\left({2\pi t}_{1}{f}_{1}\right)& \mathrm{sin}\left({2\pi t}_{1}{f}_{2}\right)& \begin{array}{cc}\cdots & \mathrm{sin}\left({2\pi t}_{1}{f}_{M}\right)\end{array}\end{array}& \begin{array}{ccc}\mathrm{ cos}\left({2\pi t}_{1}{f}_{1}\right)& \mathrm{cos}\left({2\pi t}_{1}{f}_{2}\right)& \begin{array}{cc}\cdots & \mathrm{cos}\left({2\pi t}_{1}{f}_{M}\right)\end{array}\end{array}\end{array}\\ \begin{array}{cc}\begin{array}{ccc}\mathrm{ sin}\left({2\pi t}_{2}{f}_{1}\right)& \mathrm{sin}\left({2\pi t}_{2}{f}_{2}\right)& \begin{array}{cc}\cdots & \mathrm{sin}\left({2\pi t}_{2}{f}_{M}\right)\end{array}\end{array}& \begin{array}{ccc}\mathrm{cos}\left({2\pi t}_{2}{f}_{1}\right)& \mathrm{cos}\left({2\pi t}_{2}{f}_{2}\right)& \begin{array}{cc}\cdots & \mathrm{cos}\left({2\pi t}_{2}{f}_{M}\right)\end{array}\end{array}\end{array}\end{array}\\ \vdots \\ \begin{array}{cc}\begin{array}{ccc}\mathrm{ sin}\left({2\pi t}_{n}{f}_{1}\right)& \mathrm{sin}\left({2\pi t}_{n}{f}_{2}\right)& \begin{array}{cc}\cdots & \mathrm{sin}\left({2\pi t}_{n}{f}_{M}\right)\end{array}\end{array}& \begin{array}{ccc}\mathrm{cos}\left({2\pi t}_{n}{f}_{1}\right)& \mathrm{cos}\left({2\pi t}_{n}{f}_{2}\right)& \begin{array}{cc}\cdots & \mathrm{cos}\left({2\pi t}_{n}{f}_{M}\right)\end{array}\end{array}\end{array}\end{array}\right),$$

11 $$\mathbf{X}=\left({a}_{1},{a}_{2},\cdots ,{a}_{M},{b}_{1},{b}_{2},\cdots ,{b}_{M}\right),$$

*M* is the frequency division number of the power spectrum (in this case,* M* = 100), and *f*_1_, *f*_2_, ..,* f*_*M*_ are frequency elements for the power spectrum (in this case, 0.1, 0.2, ..,10 Hz). In Eq. (11), **X** is the vector of 2* M* Fourier coefficients *a*_*i*_, *b*_*i*_ (*i* = 1, *M*) corresponding to the *i*th frequency element of the power spectrum. After Fourier transformation of Eq. (9) (Fig. 4B-2), the elements of the square root of the power spectrum for each frequency (*f*_*i*_) are given as:

12 $$\mathrm{P}\left({f}_{i}\right)=\sqrt{{a}_{i}^{2}+{b}_{i}^{2}},$$

and BS (Fig. 4B-3) is defined as:

13 $$BS=C\cdot \underset{i}{\mathrm{max}}P\left(fi\right),$$

where *C* is a constant scaling factor.

The detailed method for calculating the BS is described in the following patent application: https://patentscope2.wipo.int/search/en/detail.jsf?docId=WO2018003139.^51^

An advantage of measuring BS is the ability to apply a band-pass filter when monitoring cardiac frequency. It is therefore possible to evaluate the resistivity in an observation region peripheral to the heart. The resistivity parameter is defined as:

14 $${\text{BOM }} = {\text{ BS }}/{\text{ MBR}}_{{{\text{avg}}}} .$$

Because BS is proportional to the peak-to-peak blood flow within a given region, BOM represents the corresponding resistivity in that region.

We evaluated the systemic- and sex-dependence of the resistivity in the ONH, retinal vessels, and CHD, using previously described statistical and graphical methods for BOM analysis with a standard rubber band selection method (Fig. 4).

When assessing the relationship between BOM and BOS in the ONH, retinal vessels, the tissue surrounding the vessels and the CHD, both eyes were included.

### Calculation of BOM to assess systemic parameter- and sex-based differences in BOM values in the ONH, retinal vessels, and CHD (second aim)

To assess our second aim (systemic parameter- and sex-based differences in BOM values in the ONH, retinal vessels, and CHD), only the right eye was included. The participants were divided into two groups with a cut-off age of 50 years, to examine differences in systemic parameters between these groups.

### Application of simple linear iterative clustering (SLIC) to LSFG (Third aim)

**Figure 5:**
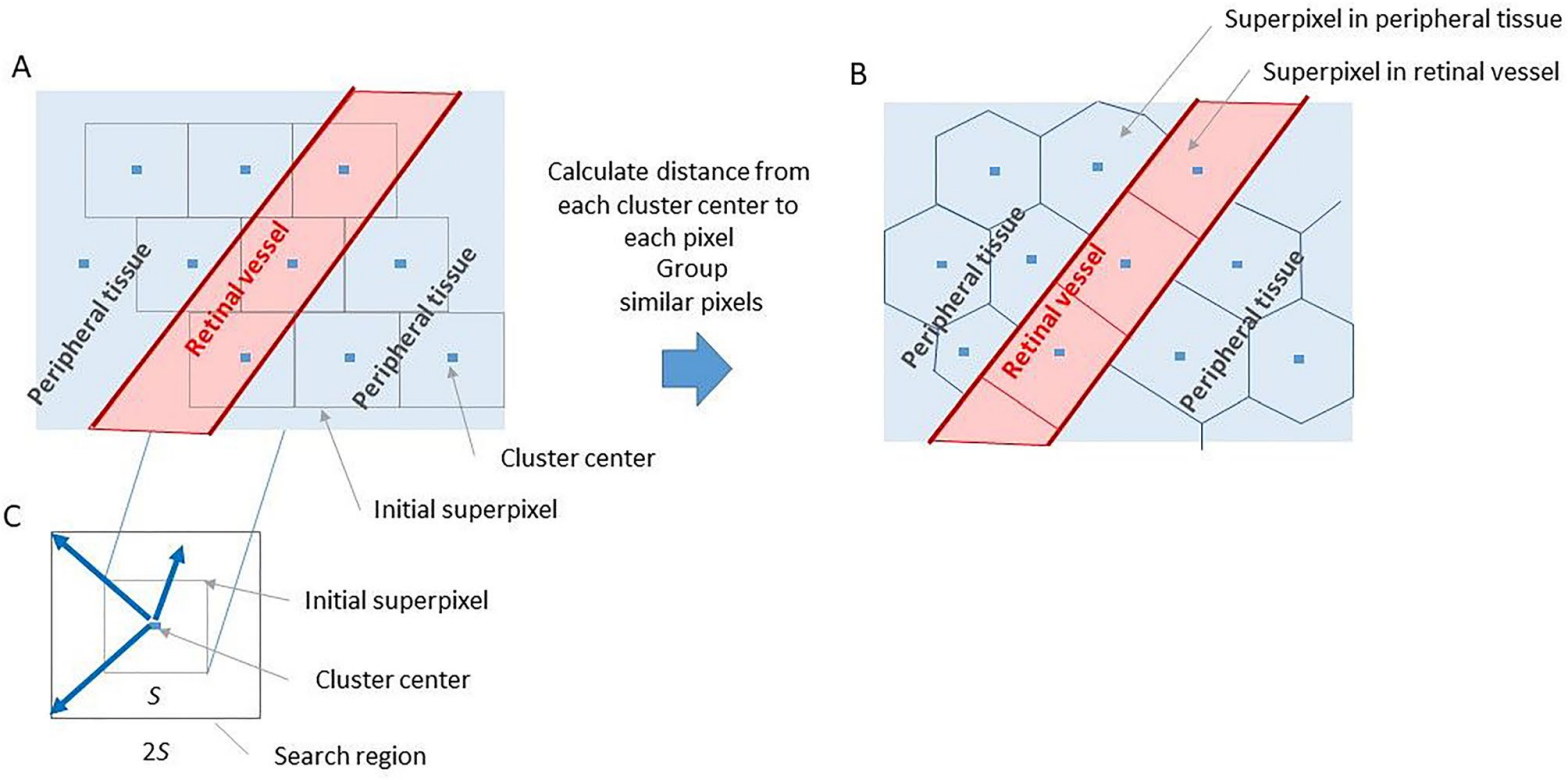
Superpixel topography in an LSFG map before and after SLIC. (**A**) Initial superpixel placement. (**B**) Final superpixel segmentation. (**C**) Initial superpixel and search region.

Recent image processing studies have identified SLIC as an excellent algorithm for clustering proximal pixels with similar colors into superpixels^52^. We proposed that this algorithm could be used to cluster pixels based on the average MBR instead of color, allowing us to reduce the computation time and speckle noise in each frame and thus allow the rapid generation of BOM maps for clinical use. Occluded vessels and those becoming occluded can be identified by their high resistivity^34^. Using the superpixel method will result in loss of spatial resolution with increasing superpixel size, and we therefore adjusted the superpixel size to preserve the resolution of large retinal vessels. Below we describe the clustering of pixels on the LSFG map into superpixels. Figure 5A,B shows the initial placement and final segmentation, respectively, of clusters on the LSFG map. The cluster centers were initially placed *S* pixels apart horizontally and vertically, with each row of clusters staggered by *S*/2 pixels. The final placement of each cluster center was determined after the superpixel grouping was completed. Figure 5C shows the initial superpixel cluster boundary containing *S* × *S* pixels and the search region containing 2*S* × 2*S* pixels, with each region enclosing a cluster center. To maintain the shape and structure of the blood vessels, we decided to use *S* = 14. The distance from the cluster center to each pixel was calculated based on LSFG parameters, such as the average MBR, and actual pixel length. The cluster to which each pixel belonged was then updated and the above procedure was repeated until no more such updates occurred. The only difference between the SLIC procedure described here and the standard SLIC method was the use of LSFG parameters instead of color to calculate the distance. Finally, the superpixels on the LSFG map were created by grouping similar pixels in each region to delineate those containing retinal vessels or peripheral tissue.

**Figure 6:**
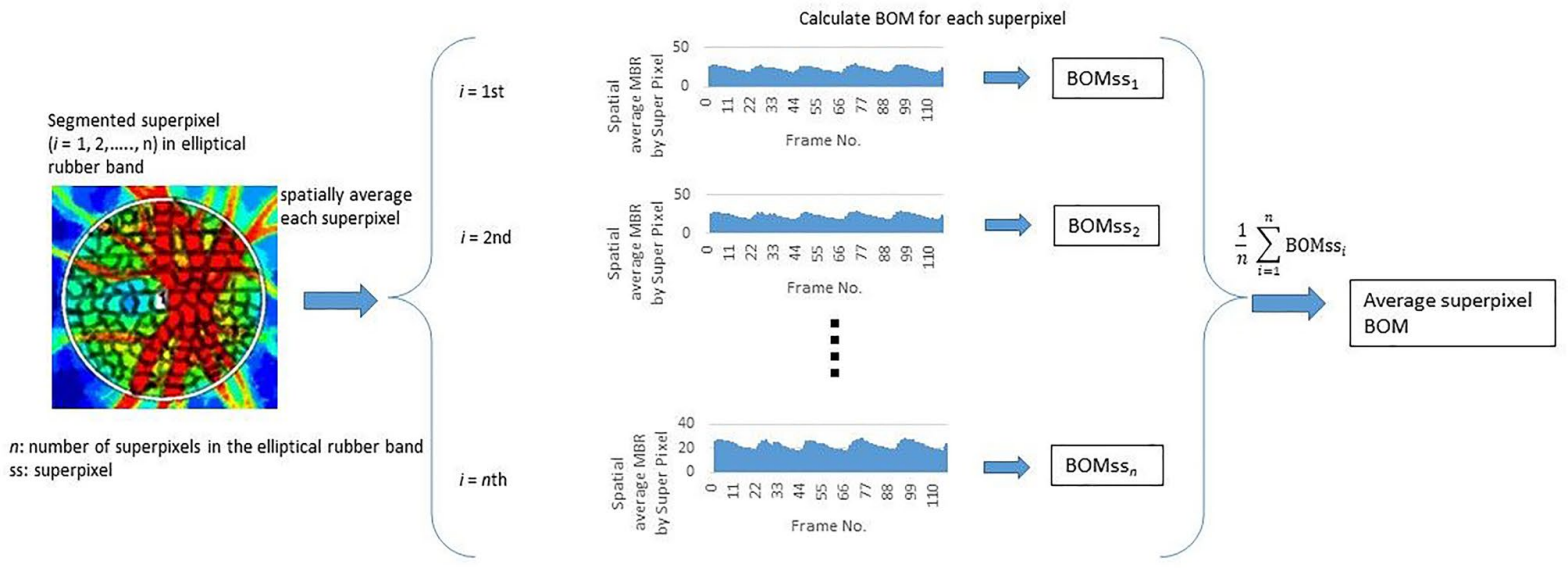
Diagram showing method for averaging BOM using the superpixel method with an elliptical rubber band placed on the ONH. The segmented superpixel figure illustrates the method rather than actual automatically segmented superpixels.

In this study, we performed pixel-averaging using the above superpixel method and by tracing an ordinary rubber band and clustering the pixels within it. In the standard rubber band method, the average BOM of the pixels enclosed within each rubber band was calculated (Fig. 4); in the superpixel method, the average BOM of the superpixels enclosed within each rubber band was calculated (Fig. 6).

LSFG data from the ONH and CHD regions in all participants were analyzed using both methods, and the correlation between the BOM output for each method was calculated using univariate regression. Additionally, the coefficients of variation of each method were calculated.

### Statistical analyses

To address the first aim, we used univariate regression analysis to assess the relationship between BOS and BOM.

To address the second aim, data for continuous variables are presented as the mean ± standard deviation. Wilcoxon’s rank sum test was used to compare systemic parameters such as body weight, and height, and sex-based differences in parameters. In addition, multiple regression analysis was performed for participants aged ≤ 50 and > 50 years, with sex, weight, height, and OPP as explanatory variables, and BOM parameters such as TCR as response variables. Each parameter was normalized to estimate the standardized partial regression coefficient. Values of *P* < 0.05 were considered to indicate statistical significance.

For the third aim, we compared BOM calculated using the superpixel method (response variable) and rubber band method (explanatory variable) by linear regression. All statistical analyses were carried out using R software (V3.5.2, www.r-project.org).

### Ethical approval

This cross-sectional study was approved by the Ethics Committee of Toho University School of Medicine (No. A16062), and all participants provided informed consent for their participation in accordance with the tenets of the Declaration of Helsinki. This study was registered in the University Hospital Medical Information Network clinical trials registry (ID: UMIN000026778).

## Data Availability

The datasets analyzed in the current study are available from the corresponding author on request.
